# Evidence of Increased Age and Sex Standardized Death Rates Among Individuals Who Accessed Opioid Agonist Treatment Before the Era of Synthetic Opioids in Ontario, Canada

**DOI:** 10.7759/cureus.19051

**Published:** 2021-10-26

**Authors:** Kristen A Morin, Frank Vojtesek, Shreedhar Acharya, John R Dabous, David C Marsh

**Affiliations:** 1 Human Sciences, Northern Ontario School of Medicine, Sudbury, CAN; 2 Office of Institutional Intelligence, Northern Ontario School of Medicine, Sudbury, CAN; 3 Curriculum Design, Northern Ontario School of Medicine, Sudbury, CAN; 4 Addiction Medicine, Institute for Clinical Evaluative Sciences (ICES) North, Sudbury, CAN; 5 Research, Innovation and International Relations, Northern Ontario School of Medicine, Sudbury, CAN

**Keywords:** standardized death rates, ontario, rural population, premature mortality, opioids use

## Abstract

Objective

The objective of this study was to evaluate age-sex standardized death rates (ASDR) from all causes from 2011 to 2015 among people who have accessed opioid agonist treatment (OAT) and compare rates living in the Northern and Southern areas of Ontario.

Methods

Routinely collected administrative health data was used to calculate crude death rates and age-sex standardized death rates (ASDRs) per 1,000,000 population of individuals who accessed OAT and compared the rates geographically from 2011 to 2015. The weighted ASDRs for each year were calculated by using the mid-year population of these regions. The rate ratios were calculated considering the base year as 2011.

Results

A total of 55,924 adults who accessed OAT were included between January 1, 2011, and December 31, 2015. The majority of patients in the cohort - 52.3% - were between 15 and 34 years old, 32.5% were female, 11.3% were in the lowest income group, 71.1% lived in Southern areas. Overall, the ASDR steadily increased during the study period and spiked in 2015. We found that among individuals who had accessed OAT, living in Southern Ontario was associated with a lower risk of all-cause mortality than those living in Northern Ontario. ASDR for Northern Ontario was 20.0 (95% confidence interval (CI)= 10.2-34.2) in 2011, and 103.5(95%CI=78.5-133.5) in 2015, which was a five-fold increase from 2011. Whereas in Southern Ontario, ASDR in 2011 was 13.8 (95% CI= 11.5-16.5), and in 2015 ASDR was 60.8 (95%CI=55.8-66.1), which was only a 4-fold increase from 2011

Conclusion

Our findings demonstrate evidence of a steadily increasing ASDR among individuals who accessed OAT with higher rates in Northern areas of the province before the era of synthetic opioids in Ontario, Canada.

## Introduction

Opioid use disorder (OUD) continues to devastate communities across North America with increasing opioid-related death rates. In Canada, as of September 2020, reports show that opioid toxicity deaths have reached their highest count since national surveillance began in 2016. By 2018, three-quarters of deaths among people who use opioids were due to synthetic opioids [1]. Mortality risk and rates have also been shown to vary according to age, gender, and geographical location [1]. For instance, in Ontario, the most populated province in Canada, Northern areas with limited access to health care services have death rates among people with OUD two times higher than urban areas [1].

Research focusing on mortality within the OUD population has often focused on crude death rates specific to opioid-related poisonings [2]. However, opioid poisoning only accounts for 21% of deaths related to opioids [2]. Other causes include homicide/suicide (19.5%), accidents, and liver disease (15%) [2]. There is a growing body of evidence suggesting that misclassification of opioid-related deaths is common because of the ambiguous circumstantial information on death certificates [3] making drawing conclusions from this information challenging. Additionally, utilizing crudes rates fails to consider influencing factors such as age and sex, where it has been shown that, on average, people with OUD are younger, and a higher proportion are male than those without OUD [4].

The literature on age and sex-adjusted death rates (ASDR) among individuals in opioid agonist treatment (OAT) in Ontario, Canada is limited. The most recent report of opioid-related standardized death rates in Ontario was conducted using data from 2004 to 2006 [5]. The data was analyzed across geographies and showed that Northern Ontario had higher rates of all-cause standardized mortality. In northern regions of Ontario, patients are subject to several barriers in accessing care. Individuals living in Northern Ontario may have difficulty accessing treatment due to the well-documented lack of primary care physicians and longer distances to travel to reach a nurse or pharmacist for observed dosing [6]. The landscape surrounding the opioid crisis has changed drastically since the last publication on standardized mortality data in 2011, including changes to policy and guidelines around opioid prescription [7], treatment and retention in OAT [8].

Reporting on ASDR specific to Ontario can provide an adequately contextualized metric that can assist in monitoring the opioid crisis by providing statistics to inform evidence-based decisions. Therefore, this study aims to evaluate ASDR for all causes among individuals who have accessed OAT in Ontario and compare these rates between Northern and Southern Ontario rates between 2011 and 2015, before the influence of the synthetic opioid crisis in Ontario [9].

## Materials and methods

Study design and setting

We conducted a retrospective cohort study of 55,924 adults who had accessed OAT in Ontario, Canada, between January 1, 2011, and December 31, 2015. We capitalized on routinely collected administrative data from publicly funded health care services to capture the escalating opioid crisis since 2011 after the spike in Oxycontin and before the era of the fentanyl and synthetic opioid crisis [9]. The data for this study were obtained through the Data Analytics Services (DAS) department at ICES. ICES is a not-for-profit research organization that gathers population-based health and social data from Ontario's publicly funded health services to generate knowledge [10]. The study data was accessed remotely using a secure server. Patient information was linked anonymously across databases using encrypted ten-digit health card numbers. The linking protocol is used routinely for health system research in Ontario [11]. The Laurentian University Research Ethics Board provided ethical approval for this study (no. 6009732). The Strengthening the Reporting of Observational Studies in Epidemiology (STROBE) guidelines were used to write this manuscript [12].

The primary cohort was created by identifying OAT patients (including those in methadone and buprenorphine/naloxone treatment) with the Ontario Drug Benefit Plan database using drug identification numbers. Only methadone in liquid formulation was included in the study since other forms of methadone are used for pain. The list of OAT medications included in the cohort creation is listed in Appendix A (Table 5). The Ontario Health Insurance Plan (OHIP) database physician billing codes including OAT monthly management codes (K682, K683, and K684), visit/consultation codes (A680 and A957) and, point of care testing codes (G040, G041, G042 or G043) were also used to identify patient in the cohort. OUD is a chronic, relapsing condition [13,14], therefore we included all patients who had accessed OAT at least once during the study period. 

We excluded all patients under 15 years of age, patients who were not eligible for OHIP, non-Ontario residents, and those with missing age, gender, and postal codes used for identification and linking across databases. We then combined patients identified from ODB, OHIP, and those identified in both databases to create the primary study cohort.

Study variables

Baseline characteristics were chosen based on a review of the literature and data availability. The following baseline summary statistics were extracted from the Registered Persons Database (RPDB) and were used to describe the study population. We included age groups (15 to 34, 35 to 64, 65+), sex (male vs female), neighbourhood-level income quintile (1 - highest, 2, 3, 4, 5) and location of residence. At the time of the study, Local Health Integration Networks (LHIN) were regional planning entities that plan and administer funding for health services across 14 defined geographic areas of Ontario. We defined Northern Ontario with LHINs 13 and 14 (North East and North West LHIN) and the remainder (i.e., LHINs 1-12) to define Southern Ontario regions. The geographical definitions have been used in previously published research [15]. The Statistics Canada Rural and Small Town definition was used to distinguish between rural and urban areas [16]. Four geographical groups were created (Northern/Rural, Northern/Urban, Southern/Rural, Southern/Urban). Comorbidity were all extracted from the OHIP database and HIV status (positive vs negative), defined by a validated ICES algorithm [17]; deep tissue infections including endocarditis (OHIP fee code 429), osteomyelitis (OHIP fee code 730) and septic arthritis (OHIP fee code 711), which have been defined in previously published research [18] and all found to be associated with injection drug use; mental health disorders (yes vs no), listed in Appendix B (Table 6). Health care use variables were counted after the patient started in OAT and included: number of emergency department visits (ED), identified using the Canadian Institute for Health Information National Ambulatory Care Reporting System (NACRS), the number of hospital admissions, identified using the Canadian Institute for Health Information Discharge Abstract Database (DAD) and primary care visits (median), identified using the Ontario Health Insurance Plan database (OHIP).

The all-cause mortality variable was defined as a death anytime during the study period, was extracted from the Registered Person's Database (RPDB).

Statistical analysis

Crude death rates for all causes per one million population were calculated by dividing the number of deaths by the mid-year population of Ontario provided by Statistics Canada [19]. We also calculated the crude death rates for Northern and Southern Ontario populations, respectively, using the mid-year population of these regions.

The rates for age and sex were adjusted using direct standardization [20] and computed 95% confidence intervals (95%CI) [21]. The Canadian population from the 2011 census was used as the standard population to calculate the standardized rate for Ontario [22]). The age-standardized death rates (ASDR) were computed for Ontario for each year of this study period. The weighted ASDRs for each year were calculated by using the mid-year population of these regions. The rate ratios were calculated considering the base year as 2011. All analysis was conducted using SAS Enterprise Guide 9.4 [23].

## Results

Patient characteristics

A total of 55,924 adults who accessed OAT between January 1, 2011, and December 31, 2015. were included. All baseline summary statistics are presented in Table 1. The majority of patients in the cohort, 52.3% were between 15 and 34 years old, 32.5% were female, 11.3% were in the lowest income group, 71.1% lived in Southern/Urban areas, 0.7% were HIV positive, 87% were diagnosed with a mental disorder, and 3% had deep tissue infections. The median number of ED visits per year was 13 (Interquartile range (IQR) 6, 25), the median number of hospitalizations was 3 (IQR = 2, 6), and the median number of primary care visits was 6 (IQR = 6, 17).

**Table 1:** Patient characteristics, comorbidities and health services use among individuals 15 years and over in OAT in Ontario (2011 to 2015). *Median number, first quartile (Q1) and third quartile (Q3). OAT: opioid agonist treatment.

Variables	N = 55,924	Proportion (%)
Age group		
15-34	29,248	52.3
35-64	25,582	45.7
65+	1,094	2.0
Sex		
Female	19,695	35.2
Male	36,229	64.8
Income Quintile (missing n= 687 )		
1 (highest)	18,408	33.3
2	12,499	22.6
3	9,972	18.1
4	8,092	14.7
5	6,266	11.3
Location of residence (missing n= 3)		
Southern Urban	43,096	77.1
Southern Rural	4,698	8.4
Northern Urban	5,294	9.5
Northern Rural	2,833	5.1
HIV Positive	411	0.7
Mental health diagnosis	48,679	87.0
Deep tissue infection	1,676	3.0
E.D. visits in one year*	13	6, 25
Hospitalizations in one year*	3	2, 6
Primary care visits in one year*	6	3, 17

Death rates by age and sex

Table 2 outlines detailed death rates by age and sex per 1,000,000. The results demonstrate that rates are highest in the youngest age group (15-34 years old). The results also demonstrate that death rates were significantly higher in the OAT cohort than the Ontario population (8 times to 20 times higher) in the 15- to 34-year-old age group. 

**Table 2:** Crude death rates by age and sex for individuals 15 years and over in OAT in Ontario (2011 to 2015). OAT: opioid agonist treatment.

2011				
Death rate (per 1,000,000)				
Sex	Age group	OAT cohort	Ontario population	Rate ratio (95% CI)
Female	15-34	2,120	294	7.2 (2.3-22.4)
Female	35-64	7,005	2,364	2.9 (2.1-4.1)
Female	65+	96,154	34,725	2.8 (1.2-6.7)
Male	15-34	4,754	625	7.6 (4.0-14.7)
Male	35-64	6,748	3,745	1.8 (1.4-2.2)
Male	65+	91,837	39,241	2.3 (1.2-4.5)
2012				
Death rate (per 1,000,000)				
Sex	Age group	OAT cohort	Ontario population	Rate ratio (95% CI)
Female	15-34	2,541	298	8.5 (3.8-19.1)
Female	35-64	6,805	2,364	2.9 (2.2-3.8)
Female	65+	55,556	35,336	1.6 (0.7-3.3)
Male	15-34	2,874	642	4.6 (2.4-8.7)
Male	35-64	8,956	3,800	2.4 (2.0-2.8
Male	65+	90,396	39,523	2.3 (1.4-3.7)
2013				
Death rate (per 1,000,000)				
Sex	Age group	OAT cohort	Ontario population	Rate ratio (95% CI)
Female	15-34	5,790	288	20.1 (12.6-32.1)
Female	35-64	8,741	2,373	2.7 (3.0-4.5)
Female	65+	51,402	34,343	1.5 (0.8-2.7)
Male	15-34	4,940	583	8.5 (5.5-13.0)
Male	35-64	8,741	3,789	2.3 (0.5-1.7)
Male	65+	35,336	38,910	0.9 (0.5-1.7)
2014				
Death rate (per 1,000,000)				
Sex	Age group	OAT cohort	Ontario population	Rate ratio (95% CI)
Female	15-34	2,681	323	8.3 (4.4-15.5)
Female	35-64	9,249	2,467	3.8 (3.1-4.5)
Female	65+	60,942	34,915	1.7 (1.1-2.7)
Male	15-34	4,082	606	6.7 (4.4-10.4)
Male	35-64	9,755	3,780	2.6 (2.3-2.9)
Male	65+	72,093	38,579	1.9 (1.3-2.7)
2015				
Death rate (per 1,000,000)				
Sex	Age group	OAT cohort	Ontario population	Rate ratio (95% CI)
Female	15-34	6,279	329	19.1 (13.0-28.1)
Female	35-64	10,007	2,441	4.1 (3.5-4.8)
Female	65+	47,431	33,189	1.4 (1.0-2.1)
Male	15-34	12,020	3,880	3.1 (2.8-3.4)
Male	35-64	57,823	37,238	1.6 (1.1-2.1)
Male	65+			

Age-standardized death rates (ASDR)

**Figure 1:**
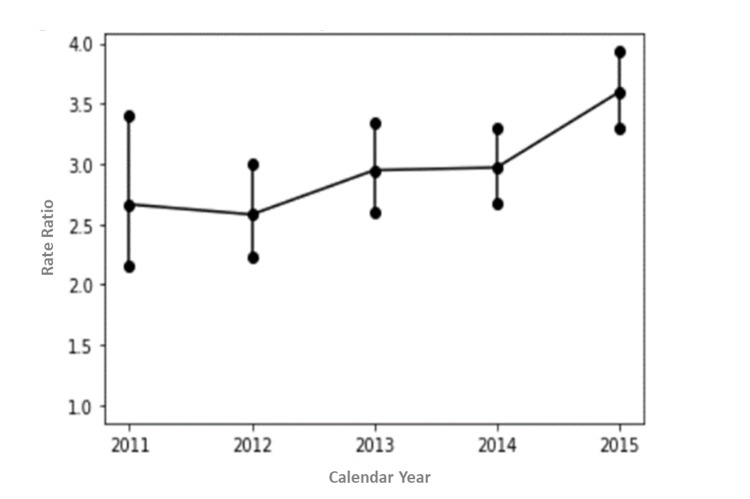
Age-sex standardized death rates (ASDR) ratio in Ontario per 1,000,000 in OAT with 95 % confidence intervals (CI). OAT: opioid agonist treatment.

As shown in Table 3, the death rate in the OAT cohort was consistently higher than the death rate in the Ontario population. Additionally, the crude death rate and ASDR steadily increased over time, spiking between 2014 and 2015. In 2011, the crude deaths rate was 6,924 per million population in the OAT cohort, compared to 7,964 in the Ontario population. In 2015 it was 11,283 per million population in the OAT cohort, compared to 8,380 in the Ontario population. In 2011, ASDR was 7,446 per 1,000,000 population in the OAT cohort compared to 2,794 in the Ontario population. In 2015, ASDR was 10,195 per 1,000,000 population in the OAT cohort to 2,835 in the Ontario population. The rate ratio was 3.6 for 2015, considering the reference year 2011. Results are graphically demonstrated in Figure 1.

**Table 3:** Age-sex standardized death rates (ASDR) for individuals 15 years and over in OAT in Ontario (2011 to 2015). OAT: opioid agonist treatment.

Calendar year	Number of deaths	Total population count	Crude death rate	Expected number of deaths	ASDR (95% CI)	Rate ratio (95% CI)
2011						2.7 (2.2-3.3)
OAT cohort	144	20,744	6,924	174,385	7,446 (5,948-9,235)	
Ontario Population	88,021	11,052,945	7,964	65,434	2,794 (2,765-2,824)	
2012						2.6 (2.2-3.3)
OAT cohort	243	30,592	7,943	170,517	7,281 (6,241-8,464)	
Ontario population	92,154	11,183,356	8,240	66,069	2,821 (2,792-2,851)	
2013						3.0 (2.6-3.3)
OAT cohort	336	39,443	8,521	192,891	8,237 (7,254-9,323)	
Ontario population	93,410	11,299,204	8,267	65,425	2,794 (2,764-2,823)	
2014						3.0 (2.7-3.3)
OAT cohort	447	47,523	9,406	197,125	8,417 (7,573-9,337)	
Ontario population	96,821	11,400,954	8,267	66,378	2,834 (2,805-2,864)	
2015						3.6 (3.3-3.9)
OAT cohort	631	55,924	11,283	238,758	10,195 (9,314-11,141)	
Ontario population	96,260	11,486,865	8,380	66,388	2,835 (2,805-2,865)	

**Figure 2:**
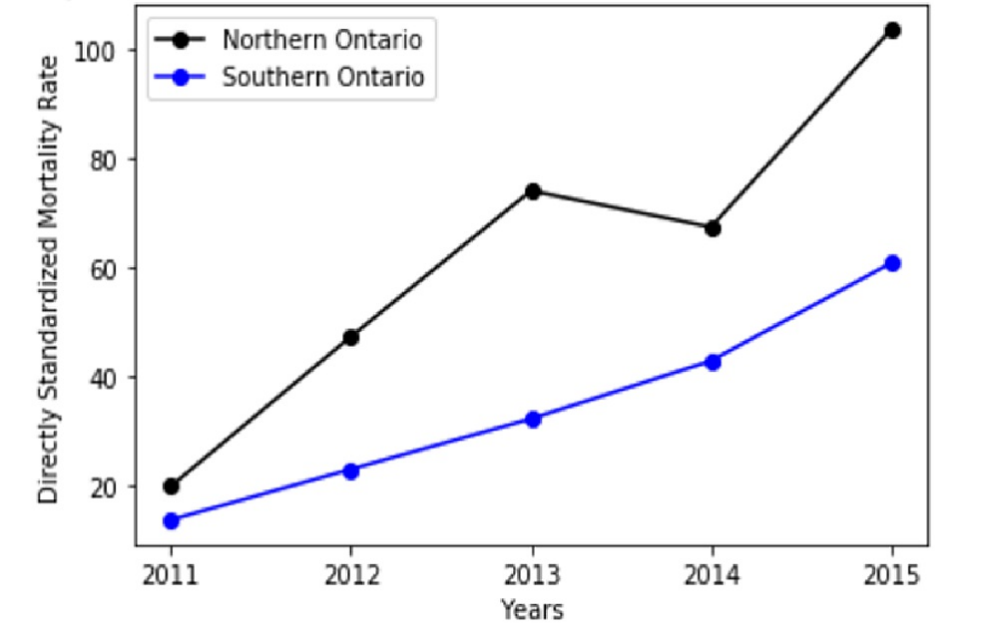
Comparison of age-standardized death rates (ASDR) in Northern and Southern Ontario for individuals in OAT (2011-2015) with 95% confidence intervals (CI). Northern Ontario: ASDR and 95% CI in 2011= 20.0 (10.2-34.2); in 2012 = 47.3 (31.2-68.3); in 2013 = 74.0 (53.2-99.7); in 2014 = 67.4 (47.6-92.1); in 2015 = 103.5 (78.5-133.5). Southern Ontario: ASDR and 95% CI in 2011= 13.8 (11.5-16.5); in 2012 = 23.1 (20.0-26.5)); in 2013 = 32.3 (28.6-36.2); in 2014 = 42.9 (38.7-47.4); in 2015 = 60.8 (55.8-66.1).

The crude death rate and ASDR, presented in Table 4, demonstrate an increase over time in both Northern and Southern Ontario. The rates of increase were higher in Northern Ontario compared to Southern Ontario. In 2011, ASDR was 20.0 (95% CI= 10.2-34.2) in Northern Ontario whereas in Southern Ontario it was 13.8 (95%CI= 11.5-16.5). In 2015, ASDR was 103.5 (95%CI= 78.5-133.5) in Northern Ontario, and in Southern Ontario it was 60.8 (95% CI= 55.8-66.1). In Northern Ontario, the rate ratio for 2015 was 5.18, considering the base year as 2011. In Southern Ontario, the rate ratio for 2015 was 4.40, considering the base year as 2011. Results are graphically demonstrated in Figure 2.

**Table 4:** Crude and age-sex adjusted death rates (ASDR) for individuals 15 years and over with OUD and rate ratio between Northern and Southern Ontario (2011 to 2015). OUD: opioid use disorder.

	Northern Ontario					Southern Ontario				
Calendar year	Number of deaths	Total population	Crude death rate	ASDR (95% CI)	Rate ratio (95% CI)	Number of deaths	Total population	Crude death rate	ASDR (95% CI)	Rate ratio (95% CI)
2011	14	681,648	20.5	20.0 (10.2-34.2)		130	10.393,359	12.5	13.8 (11.5-16.5)	
2012	28	682,199	41.0	47.3 (31.2-68.3)	2.4	215	10,521,173	22.4	23.1 (20.0-26.5)	1.7
2013	43	682,338	63.0	74.0 (53.2-99.7)	3.7	293	10,637,096	30.3	32.3 (28.6-36.2)	2.3
2014	41	680,986	60.2	67.4 (47.6-92.1)	3.4	406	10,7404,35	41.6	42.9 (38.7-47.4)	3.1
2015	65	679,116	95.7	103.5 (78.5-133.5)	5.0	566	10,828,905	57.7	60.8 (55.8-66.1)	4.4

## Discussion

This study sought to examine ASDR of individuals who accessed OAT in Ontario and compare these rates between Northern and Southern Ontario from 2011 to 2015. Drawing on a population-based cohort of 55,924 individuals, we identified that the crude death rate and ASDR in individuals who accessed OAT were consistently higher than the death rate in the Ontario population. The death rates in the youngest age group (15-34 years old) were significantly higher in the OAT cohort than the Ontario population (8 times to 20 times higher). Interestingly there were steady increases in the crude and ASDR until 2015 where rates spiked. The results also demonstrated that crude death rates and ASDR were higher in Northern Ontario compared to Southern Ontario.

The study results were consistent with other studies indicating that death rates are significantly higher among people who use opioids compared to the general population [1]. As shown in Table 2, death rates were highest in the youngest age group (15-34 years old) in the OAT cohort. This result is consistent across studies indicating that opioid use is most prevalent in the 15 to 34 age group [15]. Notably, the death rate in this age group was eight to 20 times higher in the OAT cohort compared to the Ontario population. Indicating that an otherwise healthy population is dying very prematurely. Research has shown that the effect of premature death due to opioids is so significant that it has reduced life expectancy in the United States [24] and halted increases in life expectancy in Canada for the first time in over 40 years [25]. Several reports in Ontario, including the most recent Opioid Surveillance report and the Public Health Ontario Opioid tool, have examined the incidence of opioid-related deaths across geographical settings. However, the most recent opioid-related accounts do not provide information on age and sex-specific rates. Unique to this study, with the process of age and sex standardization, the results summarize age and sex-specific information into one rate to contextualize the numbers to the Ontario population.

As shown in the trajectory plot in Figure 1, the death rate among individuals in OAT increased most significantly between 2014 and 2015. This might be representative of the beginning of the synthetic opioid era in Ontario. At the time of publication, the last published ASDR study related to opioids in Ontario examined data from 2004 to 2006 [5]. However, a 2020 study of over 55,000 OAT patients in British Columbia reported that the relative risk of death increased from 2.1 (1.8 to 2.4) to 2.6 (2.1 to 3.2) after the introduction of fentanyl in the illicit drug supply (April 1, 2012) [26]. These findings are nearly the same as those observed in the United States under the same circumstances [27]. We would expect similar results in Ontario since we know that by 2018, in Canada, three-quarters of these opioid-related deaths would involve fentanyl or other synthetic opioids [1].

We observed consistently higher ASDR in Northern Ontario among individuals who accessed OAT. Higher mortality statistics have also been published in descriptive reports and earlier studies on opioid-related mortality rates [15]. Data published for 2006 to 2008 indicated that districts in Northern Ontario were among the highest regarding mortality rates and that high rates were associated with higher opioid prescription rates [5]. Since this time, patterns of opioid use have changed due to social and economic conditions as well as the availability of opioids [28]. The trends observed in our study and across jurisdictions may reflect a change in drug use patterns from predominantly prescription opioids in the early 2000s to heroin and non-prescription use from 2009, and most recently to synthetic opioids such as fentanyl in North America [27]. More current research is needed on ASDR among people who use opioids in Ontario to examine the impact of increased availability and use of synthetic opioids.

Some limitations in the current study require consideration. First, a limitation of using administrative data to conduct research around OAT is the potential for misclassification bias. While the identification of OAT patients was based on previously published administrative data studies, the challenge is that a subset (approximately 25%) of individuals with OUD have not ever engaged in OAT [29]. Such factors have not been considered in this study. Second, the age and sex-specific death counts in Northern Ontario are small in some cases (Table 2), leading to large confidence intervals. Large confidence intervals suggest that the rates for Northern Ontario are less precise. Third, we defined our study population as individuals in OAT, meaning they had accessed OAT services at least once within the study window. However, we did not consider the impact of being actively on OAT and off OAT, nor did we consider treatment with methadone or buprenorphine/naloxone, which has been recently shown to have significant effects on death rates [26]. Fourth, this study has limited information on changing drug markets because of our study period. In Canada, the introduction of illicitly manufactured fentanyl in the drug supply has contributed to a rapidly worsening mortality rate [29]. People with OUD are at high risk of exposure to these contaminants, which are up to 10,000 times more potent than morphine. Illicitly produced fentanyl was first reported in British Columbia and Alberta in 2011. In 2017, Health Canada found fentanyl or an analogue in more than 50% of heroin samples tested by Drug Analytic Services (tested between January 2012 and September 2017) and has also started detecting it in samples methamphetamines and cocaine (2% each). Lastly, due to the use of administrative data, only variables routinely collected in health care facilities in Ontario were considered. Future studies are required to examine the impact of OAT and specifically methadone and buprenorphine/naloxone on ASDR within the context of the changing drug supply across Ontario regions.

## Conclusions

In summary, our study identified a distinct increase in ASDR in individuals who had accessed OAT before the era of increased use of synthetic opioids in Ontario. In this study, we demonstrated that there are significant differences between Northern and Southern Ontario. This finding highlights the potential value of acquiring a better understanding of the impact of geography for individuals who have accessed OAT. More current research is needed on ASDR among individuals who have accessed OAT in Ontario to examine the impact of increased availability and use of synthetic opioids.

## Appendices

**Table 5:** Opioid agonist treatment drug identification numbers.

DIN	Drug name	Route/administration
2453908	Buprenorpine HCL&Naloxone	Sublingual tables
2453916	Buprenorpine HCL&Naloxone	Sublingual tables
2295695	Buprenorpine HCL&Naloxone	Sublingual tables
2295709	Buprenorpine HCL&Naloxone	Sublingual tables
2408090	Buprenorpine HCL&Naloxone	Tablet
2408104	Buprenorpine HCL&Naloxone	Tablet
242851	Buprenorpine HCL&Naloxone	Tablet
2424878	Buprenorpine HCL&Naloxone	Tablet
2241377	Methadone HCL	Oral liquid
2244290	Methadone HCL	Oral liquid
2247694	Methadone HCL	Oral liquid
9857221	Methadone HCL	Oral liquid
9857223	Methadone HCL	Oral liquid
2394596	Methadone HCL	Oral liquid
2394618	Methadone HCL	Oral liquid
9857499	Methadone HCL	Oral liquid
9852891	Methadone HCL	Oral liquid
2481979	Methadone HCL	Oral liquid
2495872	Methadone HCL	Oral liquid
2495880	Methadone HCL	Oral liquid

**Table 6:** Mental disorder International Classification of Disease (ICD) 9 and 10 codes.

Mental disorder	ICD-10 code	ICD-9 code
Neurodevelopmental Disorders	F90 A	314
Schizophrenia Spectrum and Related Disorders	F20-F29	295
Bipolar and Related Disorders	F30-F31	296
Depressive Disorders	F32-F34	296, 311
Anxiety Disorders	F40-F41	300
Obsessive-Compulsive and Related Disorders	F42	312
Trauma and Stressor-Related Disorders	F43	308-309
Feeding and Eating Disorders	F50	307
Gender Dysphoria	F64	302
Disruptive, Impulse-Control, and Conduct Disorders	F90-F98	312-313
Personality Disorders	F60	301
